# Supplementary material for: Mediterranean UNESCO World Heritage at risk from coastal flooding and erosion due to sea-level rise
Source: Nat Commun. 2018 Oct 16;9:4161. doi: 10.1038/s41467-018-06645-9 (PMC6191433; doi:10.1038/s41467-018-06645-9)
Supplement: Supplementary file 1 — Supplementary Information [file 41467_2018_6645_MOESM1_ESM.pdf]

SUPPLEMENTARY INFORMATION

Mediterranean UNESCO World Heritage at risk from coastal flooding and erosion due to sea-level rise

Reimann et al.

PLEASE NOTE:

This document includes all supplementary information provided in figures and tables. For the supplementary data please see the following files:

**Document name**	**Description**
Supplementary_Data_1	Results of the flood risk calculations
Supplementary_Data_2	Results of the erosion risk calculations
Supplementary_Data_readme	Description of the variables included in Supplementary Data 1 and 2

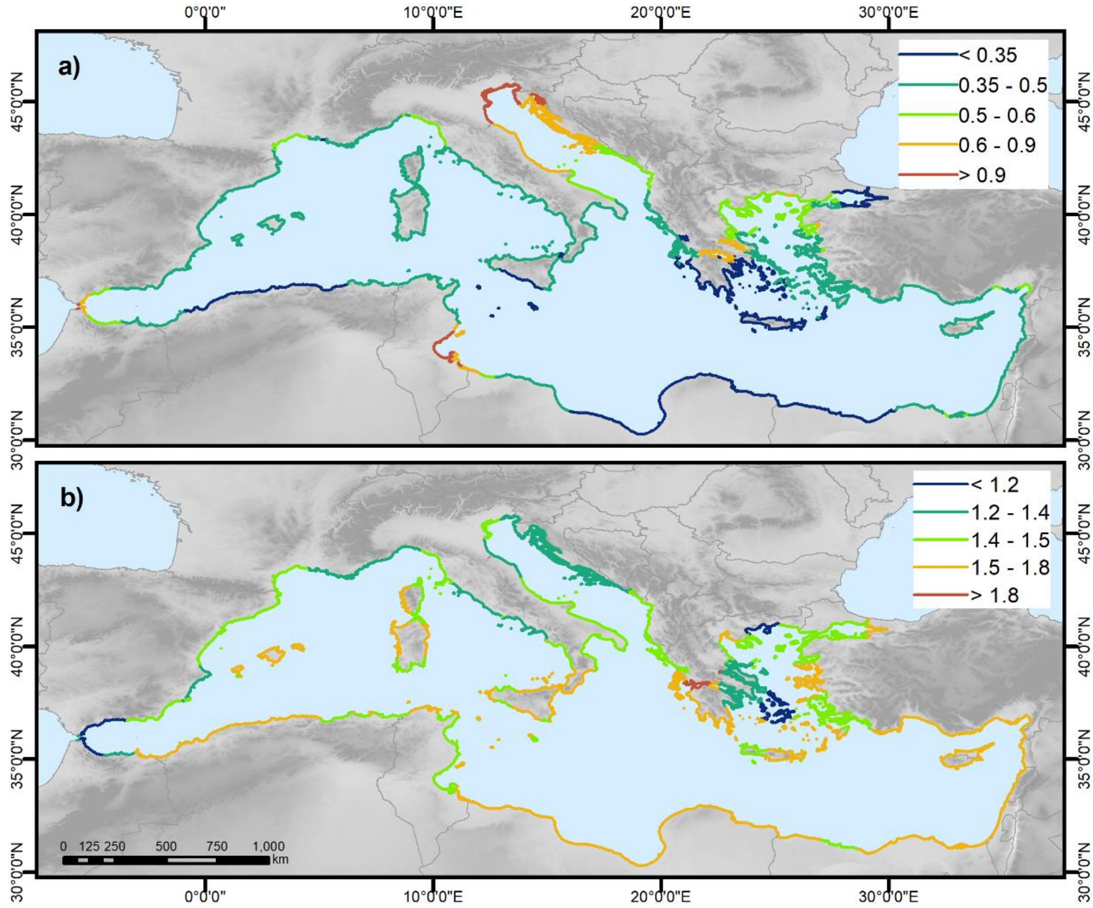

Supplementary Figure 1 Spatial patterns of the extreme sea level components storm surge and sea-level rise. **a)** 100-year storm surge (in m) taken from the Mediterranean Coastal Database[2], **b)** regional sea-level rise (in m) in 2100 under the high-end scenario based on Kopp et al. (2017)[4]

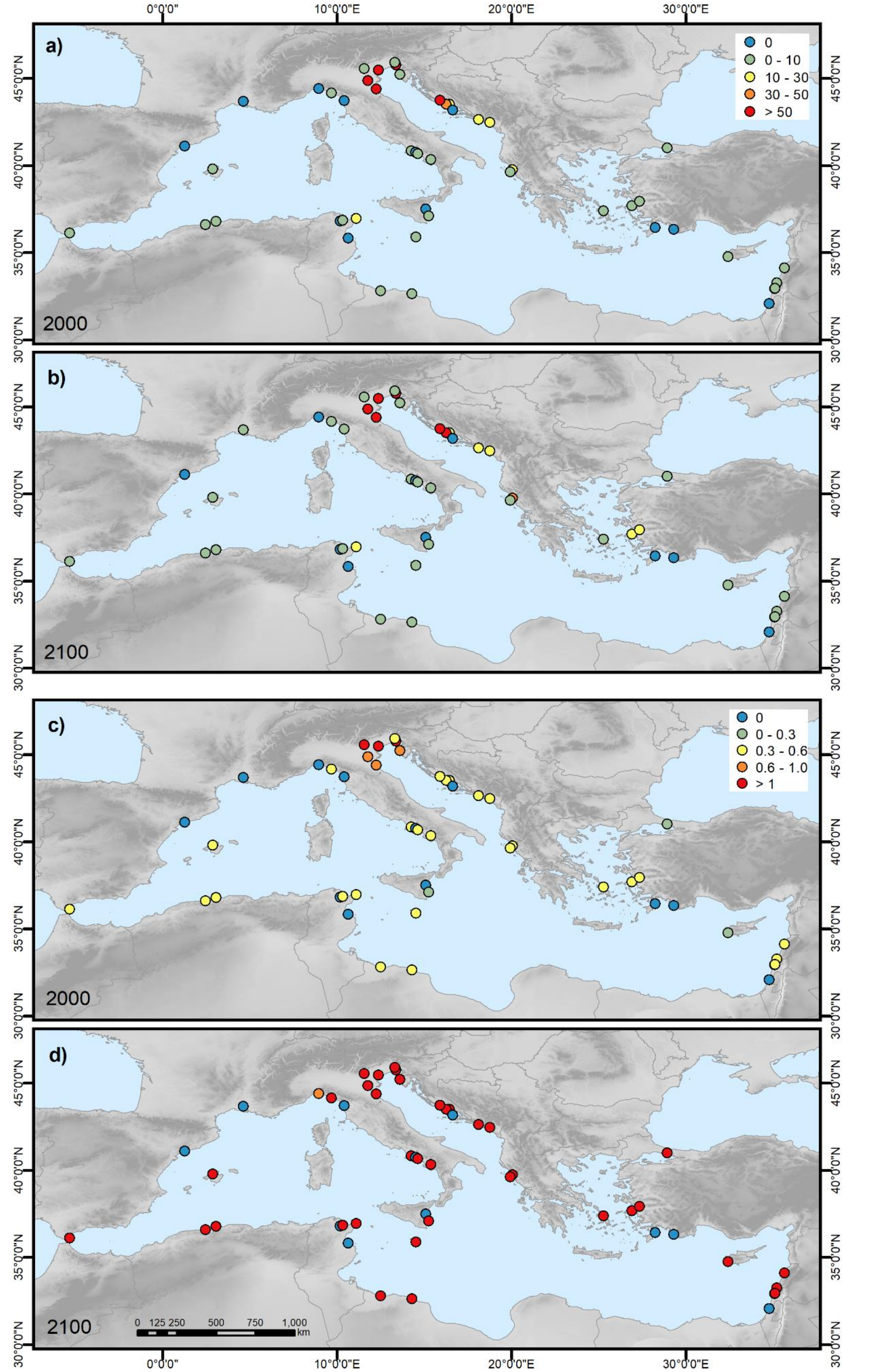

Supplementary Figure 2 Characteristics of the flood risk indicators flood area and flood depth at each World Heritage site under current and future conditions. **a)** and **b)** area flooded (in %) in the base year 2000 (a) and in 2100 under the high-end sea-level rise scenario (b), **c)** and **d)** maximum flood depth (in m) in 2000 (c) and in 2100 under the high-end sea-level rise scenario (d)

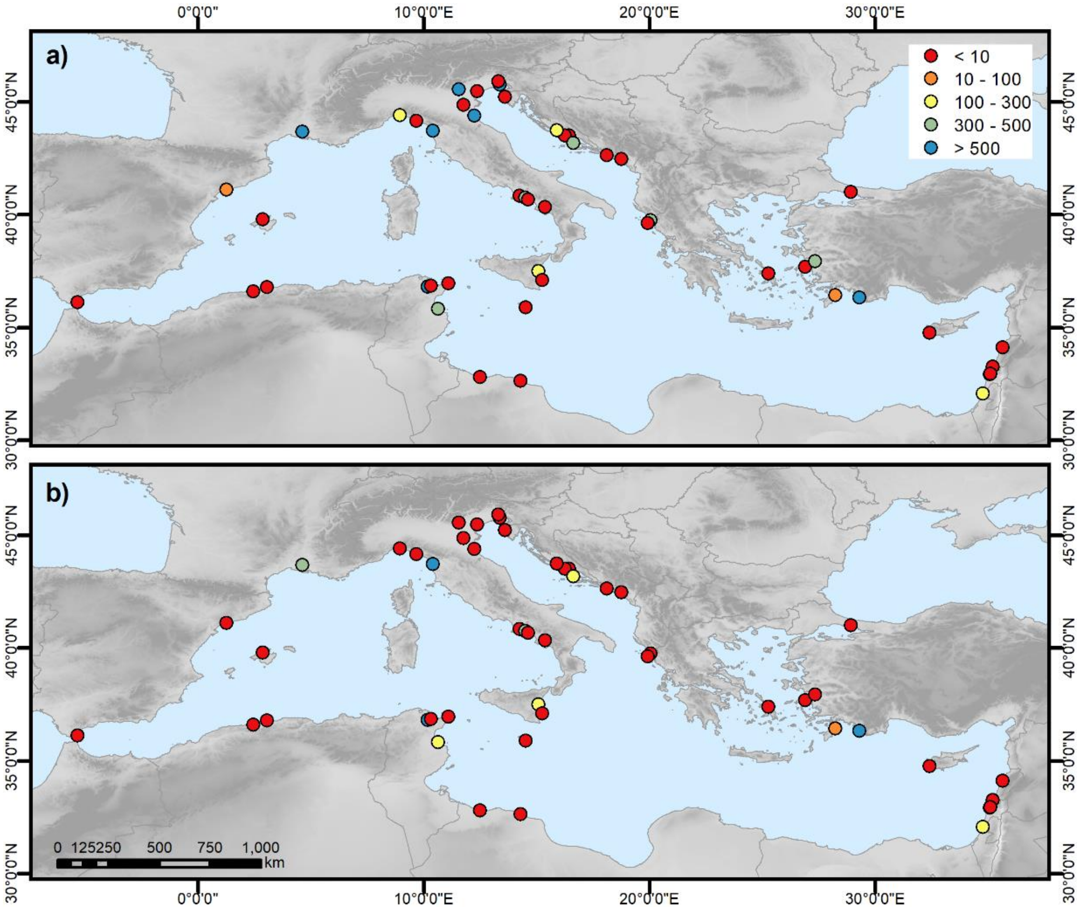

Supplementary Figure 3 Characteristics of the erosion risk indicator distance from the coastline (in m) at each World Heritage sites under current and future conditions. **a)** in 2000, **b)** in 2100 under the high-end sea-level rise scenario

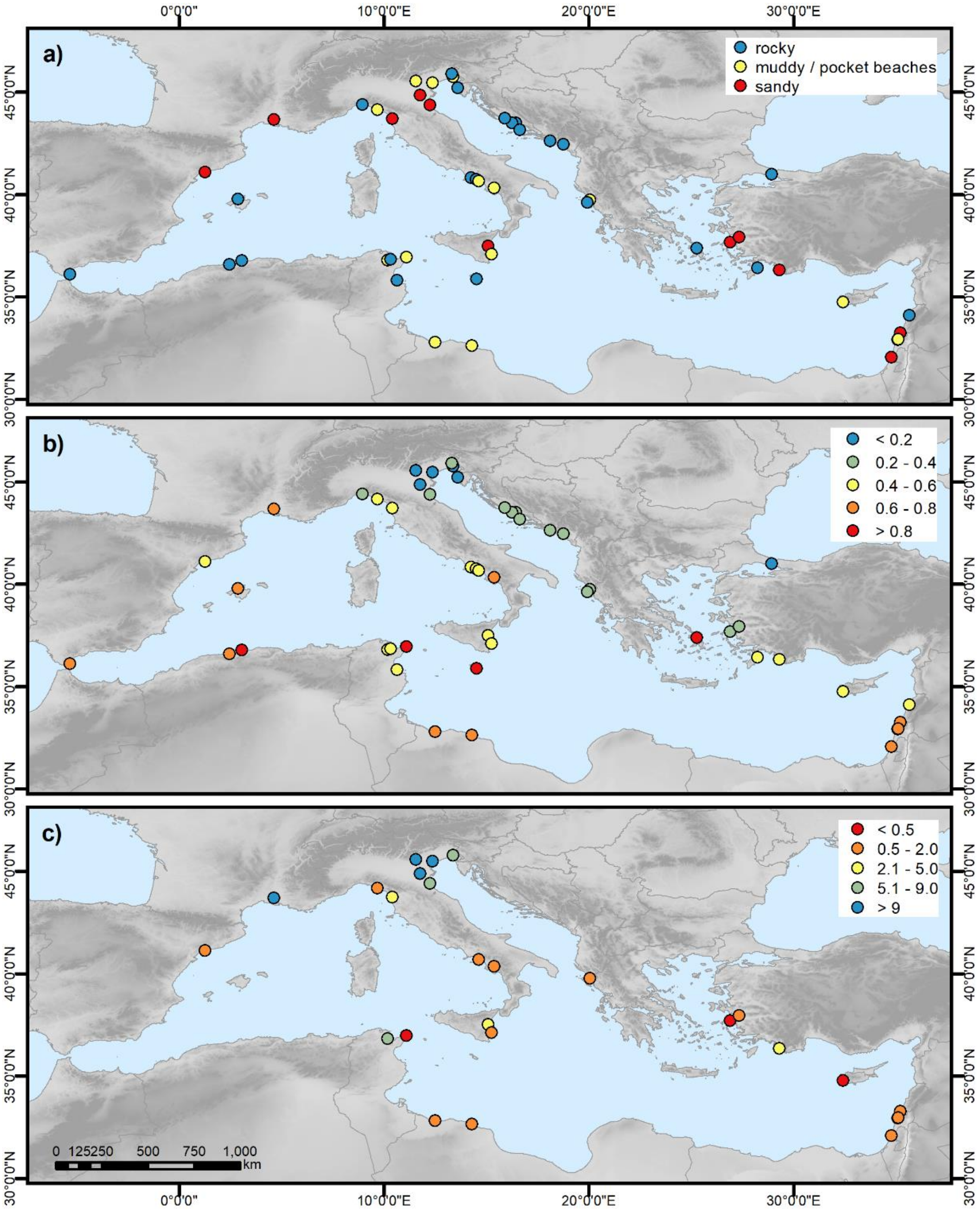

Supplementary Figure 4 Characteristics of the static erosion risk indicators at each World Heritage site. **a)** coastal material, **b)** mean wave height (in m), **c)** sediment supply (in mg l^{-1}) given for coastal materials other than rocky.

Supplementary Table 1 Attributes of the corrected Mediterranean UNESCO World Heritage site data

Attribute name	Description	original dataset[a]	added[b]
unique_id	unique ID of each serial site		x
id_no	ID of main site	x	
site_id	ID of serial nomination		x
name_seria	Name of serial nomination		x
name_en	Name of main site in English	x	
name_fr	Name of main site in French	x	
date_inscr	Date when it was inscribed in the list	x	
sec_date	Date when changes have been made (e.g. adjusting boundary)	x	
danger_lis	Date when it was put on the danger list (if applicable)	x	
longitude	X coordinate of center point in decimal degrees	x	x*
latitude	Y coordinate of center point in decimal degrees	x	x*
area_ha	Area of site in hectares (excl. buffer zone) (-9999 = not given)	x	x*
area_ha1	Area of site in hectares (excl. buffer zone) as calculated based on the WHS polygons produced		x
C1 – C6	Criteria of Outstanding Universal Value (OUV) (1/0)	x	
criteria_t	OUV criteria in text	x	
category	Cultural (or natural)	x	
category_s	C for cultural	x	
states_en	Name of the country (countries) in English	x	
states_fr	Name of the country (countries) in French	x	
region_en	Name of the region in English	x	
region_fr	Name of the region in French	x	
iso_code	2-digit country code	x	
undp_code	3-digit country code of the UNDP (United Nations Development Programme)	x	
transbound	Cross-border site (1/0)	x	
no_serial	Number of serial sites		x
her_type1<br>her_type2	Heritage type based on ICOMOS (2011)[1] and Daly (2014)[3]		x

her_type3	1 = cultural landscape<br>2 = built heritage/architecture/historic urban center<br>3 = archaeological remains<br>4 = single monument		
buffer_ha	Buffer area in hectares (-9999 = not given)		x
srtm_min	Lowest site elevation in the SRTM90 DEM[5,6]		x
srtm_max	Highest site elevation in the SRTM90 DEM		x
srtm_mean	Mean site elevation in the SRTM90 DEM		x
p_lecz	Percent of site located in the LECZ		x
ur_grump	Location of WHS in urban areas based on the GRUMP urban extents grid[7] (1/0)		x
ur_mod_buf	Location of WHS in urban areas based on the MODIS urban extents grid[8] with a 500 m buffer (1/0)		x
ur_joined	Combination of ur_grump and ur_mod_buf based on Google Earth™ satellite imagery		x
dist_MCD	Distance from the coast [m] based on the Mediterranean coastal database (MCD)[2]		x
dist_gshhs	Distance from the coast [m] based on the global self-consistent, hierarchical, shoreline database (GSHHS) version 2.3.7 by Wessel et al. 1996[9]		x
dist_join	Combination of dist_MCD and dist_gshhs based on Google Earth™ satellite imagery		x

= included in the serial site dataset only

= included in the main site dataset only

[a] taken over from the original World Heritage List data of 2018[10]

[b] added to the original World Heritage List data with the help of the data sources stated in the description

* modified from original World Heritage List data

Supplementary Table 2 Data used

Variable	Indicator(s)	Reference
Coastal World Heritage	World Heritage sites 2018	UNESCO (2018)[10]
Elevation	Shuttle Radar Topography Mission (SRTM) DEM	Farr et al. (2007)[6], Jarvis et al. (2008)[5]
Flood risk		
Sea-level rise scenarios	RCP2.6, RCP4.5, RCP8.5 (50th percentile)<br>High-end (RCP8.5, 95th percentile)*	Kopp et al. (2017)[4]
Storm surge	100-year surge height	Wolff et al. (2018)[2], based on Muis et al. (2016)[11]
Mean dynamic ocean topography (MDT)	Used to reference the surge heights to the EGM96 geoid	Wolff et al. (2018)[2], based on Rio et al. (2014)[12]
Erosion risk		
World Heritage distance from the coastline	Mediterranean Coastal Database (MCD);<br>Global self-consistent, hierarchical, shoreline database (GSHHS) version 2.3.7	Wolff et al. (2018)[2]<br>Wessel et al. (1996)[9]
Erodibility	Coastal material	Wolff et al. (2018)[2]
Waves	Mean wave height	Wolff et al. (2018)[2]
Sediment supply	Total suspended matter	Schuerch et al. (in press)[13], based on data of the GlobColour project[14]

* We found the sea-level rise projections of the high-end scenario in 2100 to be lower than those of 2090 at a number of grid points, which we considered to be implausible due to the fact that the projections post-2100 continue to increase in an accelerating manner. Therefore we have calculated the mean of the sea-level rise growth rates between the years 2080-2090 and 2110-2120 and added it to the projection of 2090 to adjust the projection of 2100.

Supplementary References

1. International Council on Monuments and Sites (ICOMOS). Guidance on Heritage Impact Assessments for Cultural World Heritage Properties. Available at https://www.icomos.org/world_heritage/HIA_20110201.pdf (2011).
2. Wolff, C. *et al.* A Mediterranean coastal database for assessing the impacts of sea-level rise and associated hazards. *Scientific Data* **5,** 180044 (2018).
3. Daly, C. A Framework for Assessing the Vulnerability of Archaeological Sites to Climate Change. Theory, Development, and Application. *Conservation and Management of Archaeological Sites* **16,** 268–282 (2014).
4. Kopp, R.E. *et al.* Evolving Understanding of Antarctic Ice-Sheet Physics and Ambiguity in Probabilistic Sea-Level Projections. *Earth's Future* **5,** 1217–1233 (2017).
5. Jarvis, A., Reuter, H.I., Nelson, A. & Guevara, E. Hole-filled SRTM for the globe Version 4, available from the CGIAR-CSI SRTM 90m Database. Available at http://srtm.csi.cgiar.org (2008).
6. Farr, T.G. *et al.* The Shuttle Radar Topography Mission. *Rev. Geophys.* **45** (2007).
7. Center for International Earth Science Information Network - Columbia University (CIESIN), International Food Policy Research Institute (IFPRI), The World Bank & Centro Internacional de Agricultura Tropical (CIAT). *Global Rural-Urban Mapping Project, Version 1 (GRUMPv1): Urban Extents Grid* (NASA Socioeconomic Data and Applications Center (SEDAC), Palisades, NY, 2011).
8. Schneider, A., Friedl, M.A. & Potere, D. A new map of global urban extent from MODIS satellite data. *Environ. Res. Lett.* **4,** 44003 (2009).
9. Wessel, P. & Smith, W.H.F. A global, self-consistent, hierarchical, high-resolution shoreline database. *J. Geophys. Res.* **101,** 8741–8743 (1996).
10. UNESCO World Heritage Centre. World Heritage List. Available at http://whc.unesco.org/en/list/ (2018).
11. Muis, S., Verlaan, M., Winsemius, H.C., Aerts, J.C.J.H. & Ward, P.J. A global reanalysis of storm surges and extreme sea levels. *Nature communications* **7,** 11969 (2016).
12. Rio, M.-H., Mulet, S. & Picot, N. Beyond GOCE for the ocean circulation estimate. Synergetic use of altimetry, gravimetry, and in situ data provides new insight into geostrophic and Ekman currents. *Geophys. Res. Lett.* **41,** 8918–8925 (2014).
13. Schuerch, M. *et al.* Future response of global coastal wetlands to sea level rise. *Nature* (in press).
14. Doerffer, R. & Schiller, H. The MERIS Case 2 water algorithm. *International Journal of Remote Sensing* **28,** 517–535 (2010).
