# Supplementary material for: Mediterranean UNESCO World Heritage at risk from coastal flooding and erosion due to sea-level rise
Source: Nat Commun. 2018 Oct 16;9:4161. doi: 10.1038/s41467-018-06645-9 (PMC6191433; doi:10.1038/s41467-018-06645-9)
Supplement: Supplementary file 3 — Description of Additional Supplementary Files [file 41467_2018_6645_MOESM3_ESM.pdf]

Variables included in Supplementary Data 1 and Supplementary Data 2
Please see associated article (+ Supplementary Information) for further details

VARIABLE	DESCRIPTION
id_no	official UNESCO World Heritage site ID (see Figure 1 in the article)
year	2000-2100 in 10-year time steps
scenario	sea-level rise scenario based on RCP2.6, RCP4.5, RCP8.5 and one high-end scenario

Flood risk (Supplementary Data 1)

area	area of World Heritage site flooded (in %)
depth	maximum flood depth (in m)
index	flood risk index

Erosion risk (Supplementary Data 2)

distance	shortest distance of World Heritage site from the coastline (in m)
material	coastal material
mWH	mean wave height (in m)
sedisup	sediment supply (in mg/l)
index	erosion risk index
